# Multi-center study on patient selection for and the oncologic safety of intraoperative radiotherapy (IORT) with the Xoft Axxent® eBx® System for the management of early stage breast cancer in Taiwan

**DOI:** 10.1371/journal.pone.0185876

**Published:** 2017-11-02

**Authors:** Hung-Wen Lai, Liang-Chih Liu, Fu Ouyang, Chung-Chin Yao, Hsiang-Chun Jan, Ya-Herng Chang, Chi-Wen Tu, Dar-Ren Chen, Tsui-Fen Cheng, Yen-Dun Tzeng, Huan-Ming Hsu, Ming-Hsin Yeh, Yao-Chung Wu, Po-Sheng Yang, Hung-Bun Lam, Ming-Feng Hou, Fang-Ming Chen

**Affiliations:** 1 Endoscopy & Oncoplastic Breast Surgery Center, Changhua Christian Hospital, Changhua, Taiwan; 2 Comprehensive breast cancer center, Changhua Christian Hospital, Changhua, Taiwan; 3 School of Medicine, National Yang Ming University, Taipei, Taiwan; 4 Department of Surgery, China Medical University Hospital, Taichung, Taiwan; 5 College of medicine, China Medical University, Taichung, Taiwan; 6 Kaohsiung Municipal Ta-Tung Hospital, Kaohsiung, Taiwan; 7 Breast Division, Department of Surgery, Kaohsiung Medical University Hospital, Kaohsiung, Taiwan; 8 Kaohsiung Medical University, Kaohsiung, Taiwan; 9 Department of Surgery, Chung Shan Medical University Hospital, Taichung, Taiwan; 10 School of Medicine, Chung Shan Medical University, Taichung, Taiwan; 11 Cardinal Tien Hospital, Taipei, Taiwan; 12 Department of General Surgery, Ditmanson Medical Foundation Chia-Yi Christian Hospital, Chia-Yi, Taiwan; 13 Shin Kong Wu Ho-Su Memorial Hospital, Taipei, Taiwan; 14 Division of General Surgery, Department of Surgery, Kaohsiung Veterans General Hospital, Kaohsiung, Taiwan; 15 Department of Surgery, Tri-Service General Hospital, Songshan branch, Taipei, Taiwan; 16 Department of Surgery, Mackay Memorial Hospital, Taipei, Taiwan; 17 Department of Medicine, Mackay Medical College, New Taipei, Taiwan; 18 Department of Surgery, Kaohsiung Municipal Hsiao-Kang Hospital, Kaohsiung, Taiwan; University of North Carolina at Chapel Hill School of Medicine, UNITED STATES

## Abstract

**Background:**

In this multi-center study, we report the patient selection criteria for and preliminary oncologic outcomes associated with intraoperative radiotherapy (IORT) delivered by the Xoft Axxent® eBx® system for early-stage breast cancer in Taiwan.

**Methods:**

Patients with early breast cancer in Taiwan received breast conserving surgery and received IORT with Xoft Axxent® eBx® System during 2013–2015 was search from database of Taiwan IORT study cooperative group (T-IORTSCG). Patients’ clinicopathologic characteristics and early post-operative results were collected and reported.

**Results:**

During the study period, 26 hospitals in Taiwan performed a total of 261 Xoft IORT procedures for breast cancer. The mean age of them was 52.9 ± 9.8 years (37–72), and tumor size was 1.5 ± 0.8 cm (0.1–4.2 cm) for invasive cancer and 1.2 ± 0.8 cm (range, 0.2–3.0 cm) for ductal carcinoma in situ (DCIS) lesions. Lymph node metastasis was found in 6 (2.3%) patients. The patients received IORT in Taiwan differed markedly from those used in the ELIOT and TARGIT-A studies. Specifically, patients selected for IORT in Taiwan tended to be younger, their tumors tended to be larger and the prevalence of lymph node metastasis tended to be lower. Among these 261 patients, 8 (3.1%) patients required whole breast radiotherapy. During a mean follow up of 15.6 months, locoregional recurrence was observed in 2 (0.8%) patients.

**Conclusion:**

In real world experience, patients received IORT differed quite significantly with criteria formulated by trials. The preliminary results of IORT in Taiwan showed it is well acceptable by patients and clinicians.

## Introduction

Breast conserving surgery (BCS) followed by whole-breast external beam radiotherapy (WBRT) has become the mainstay of surgical treatment for early-stage breast cancer [1, 2]. WBRT reduces the likelihood of local recurrence in the conserved breast and lowers the risk of death due to breast cancer [3]. However, conventional WBRT, which is administered daily over a 6- to 7-week period, precludes a significant proportion of women from receiving the full course of radiation treatment [4–6]. Intraoperative radiotherapy (IORT), in which postoperative whole-breast irradiation is substituted for one session of radiotherapy with the same equivalent dose during surgery, solves this problem by allowing for treatment to be completed on the same day. Recent trials such as electron intraoperative radiotherapy versus external radiotherapy for early breast cancer (ELIOT trial) [7] and targeted intraoperative radiotherapy versus whole breast radiotherapy for breast cancer (TARGIT-A trial) [8, 9] have demonstrated that IORT in some selected groups of low-risk early breast cancer patients results in acceptable outcomes and could, therefore, serve as an alternative to conventional WBRT.

IORT using the Axxent electronic brachytherapy (eBX) system (Xoft, Inc., San Jose, CA) for the treatment of breast cancer is a relative new method of delivering accelerated partial breast irradiation (APBI) that aims to replace WBRT in selected women suitable for BCS. The one-year results of a trial utilizing eBX to deliver 5-day APBI treatment have shown it to be an effective alternative method with minimal acute side-effects [10]. Another single-institution trial also found that delivery of IORT via the eBX system was efficacious and safe for women with early-stage breast cancer [11].

The Xoft IORT using the Axxent electronic brachytherapy (eBX) system was introduced for the treatment of breast cancer in Taiwan in May of 2012. The Taiwan IORT study cooperative group (T-IORTSCG) was established to monitor the effectiveness of and clinical outcomes associated with the Xoft Axxent® eBx® IORT delivery system for the management of early-stage breast cancer in Taiwan. In this multi-center study, we report the patient selection criteria for and preliminary oncologic outcomes associated with this new type of radiotherapy.

## Materials and methods

### Patients

In this study, we collected clinicopathologic data from the T-IORTSCG database on patients who underwent IORT for breast cancer during the period January 2013 to December 2015 at T-IORTSCG-affiliated institutions. The clinicopathologic data collected from the database included patient characteristics, type of surgery, type and dose of IORT, recurrence, and survival status at the most recent follow-up. The data gathered from the database covered more than 95% of the IORT procedures performed in Taiwan during the study period and therefore can be interpreted as representing the status of IORT in Taiwan. All data were collected by chart review by a specially trained nurse and confirmed by the principle investigator (HWL). The study was approved by the Institutional Review Board of the Changhua Christian Hospital (CCH IRB No.: 151004). Due to the retrospective and chart review nature of this study, the ethics committees (IRB) in our hospital decided no written or verbal informed consent was needed by the participants. Patient records/information was anonymized and de-identified prior to analysis.

### Patient selection for IORT

A preoperative tissue diagnosis of cancer of the breast was required prior to the operation. Pre-operative mammography and sonography were used in all patients to determine their eligibility for IORT. Magnetic resonance imaging (MRI) was used optionally for selection of patients’ suitability for IORT. Liver sonography, chest X ray, and whole body bone scan were used in all patients to exclude the possibility of distant metastasis.

The inclusion and exclusion criteria were based on those reported previously [7–9, 11–14]. The inclusion criteria for patients suitable for IORT were unifocal tumors of less than 3 cm, no evidence of lymph node involvement, the presence of invasive ductal carcinoma (IDC) or ductal carcinoma in situ (DCIS), and a minimum age of 45 years. Contraindications for IORT included inflammatory breast cancer, breast cancer with chest wall or skin invasion, locally advanced breast cancer, breast cancer with extensive axillary lymph node metastasis (stage IIIA or later), and severe co-morbid conditions such as heart disease, renal failure, liver dysfunction, or poor performance status as assessed by primary care physicians. All patients underwent extensive preoperative counseling by the surgeon and the radiation oncologist. Radiation treatment options were explained to the patients, including standard WBRT as well as IORT.

### Surgical treatment and eBX IORT procedures

**Fig 1:**
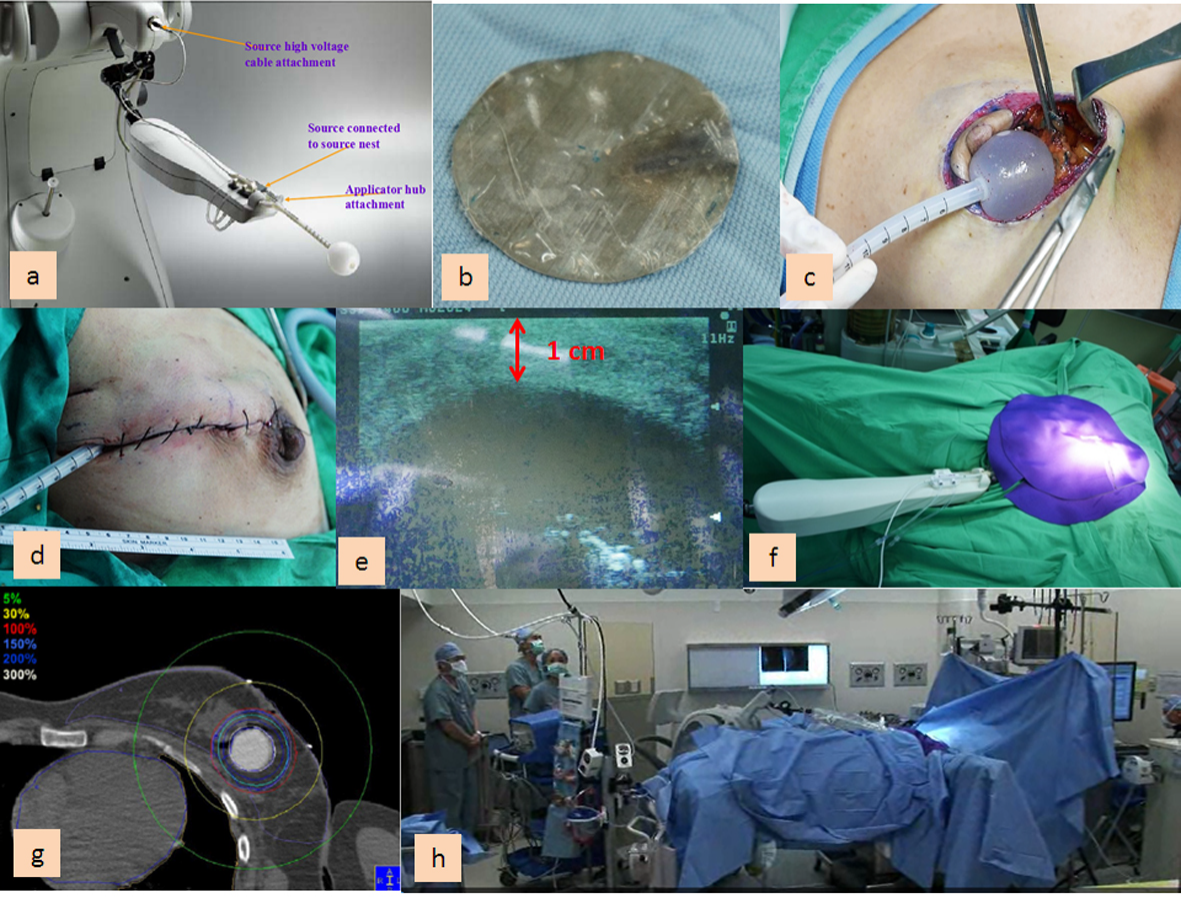
Procedures for patients received intra-operative radiotherapy with the Xoft Axxent® eBx® delivery system. (a) The eBX system consists of a balloon applicator, a 50-kV source, and a mobile, highly portable controller unit that can be easily transported to any treatment room or standard operating room. (b) The chest wall shield was placed temporarily into the cavity for the duration of radiation treatment to protect the underlying heart, ribs, and lungs from scattered radiation. (c) A balloon-like cavity evaluation device was then placed through a lateral stab wound incision or directly into the wound and filled to a desired volume of 30–75 cc, based on the radiation treatment plan. (d) Once the cavity volume was determined, an appropriate size of eBX balloon was opened up and inserted into the cavity. Multiple retention-type sutures were used to maintain the balloon-to-tissue apposition and to temporarily close the lumpectomy cavity around the balloon. (e) Balloon-to-tissue conformity was assessed by intraoperative ultrasonography to ensure that the target volume did not contain air or fluid. Intraoperative ultrasonography was then used to confirm that there was a distance of at least 1 cm between the balloon and skin to reduce the risk of radionecrosis. (f) A FlexiShieldTM (FS; Xoft, Inc., San Jose, CA) was placed over the breast to minimize transmission of radiation to the patient and hospital staff. (g) The radiation source was inserted into the balloon and radiation therapy was initiated. A planned dose of 20 Gy to the balloon surface was delivered over an average of 8–15 mins. (h) During treatment, the surgeon, radiation oncologists, anesthesiologist, and other essential operating room personnel wore standard lead aprons and /or stood behind a portable radiation shield in the operating suite. The medical staffs could also leave the operation room and observe the monitors during the Xoft IORT treatment.

The protocol for carrying out IORT via the Xoft Axxent® eBx® delivery system is described in detail by Ivanov et al. [11], and illustrated in Fig 1. In brief, sentinel lymph node biopsy (SLNB) [15] was usually done first. Then BCS was performed, and intra-operative frozen section for margin status analysis was not mandatory. After excision of the tumor and a margin of healthy tissue, breast tissue dissection was carried down to the level of the pectoralis fascia in preparation for IORT. The tumor bed was mobilized to ensure that there was a distance of at least 10 mm between the surface of the applicator and the skin in order to reduce the risk of radionecrosis (Fig 1). The radiation source was inserted into the balloon and radiation therapy was initiated. A planned dose of 20 Gy to the balloon surface was delivered over an average of 8–15 mins. After radiation treatment, the retention sutures, the eBX balloon and the lead shield were removed. The lumpectomy cavity was irrigated and closed in a standard manner or an oncoplastic technique was performed to prevent parenchyma defects [16, 17].

### Post-operative systemic therapy and follow-up

Postoperative adjuvant hormone therapy, chemotherapy and radiotherapy were given to patients according to current breast cancer treatment guidelines [18, 19]. The rate of positive surgical margin involvement, locoregional recurrence, distant metastasis, and mortality were recorded and analyzed. In current study, the definition of negative margin was no tumor on ink. Total incidence of recurrence or death due to breast cancer was ascertained at the most recent follow-up, which ended on July 2016.

### Statistical analyses

Data are expressed as mean ± standard deviation (SD) for continuous variables. Categorical variables were tested by the chi-square test when appropriate. Differences in means of continuous variables were tested by the Student’s t test. All p values are two-tailed; a p value of less than 0.05 was considered to indicate statistical significance. All statistical analyses were performed with the statistical package SPSS for Windows (Version 19.0, SPSS, Chicago).

## Results

During the study period, a total of 261 patients with breast cancer received IORT procedures with the Xoft Axxent® eBx® system in Taiwan. The mean age of the patients was 52.9 ± 9.8 years. The mean tumor size was 1.5 ± 0.8 cm (0.1–4.2 cm) for invasive cancer and 1.2 ± 0.8 cm (0.15–3.0 cm) for DCIS lesions. Most (95.8%) of them were node negative breast cancer patients (Table 1).

**Table 1:** Clinicopathologic characteristics of patients selected for Xoft IORT in Taiwan.

N = 261		
Age	52.9 ± 9.8 (37–72)	
<45	43	(16.5%)
45–60	147	(56.3%)
>60	56	(21.5%)
NA	15	(5.7%)
Tumor Size (in situ, cm)	n = 41	1.2 ± 0.8 (0.15–3.0)
Tumor Size (invasive, cm)	n = 220	1.5 ± 0.8 (0.1–4.2)
T1a	18	(8.2%)
T1b	40	(18.2%)
T1c	108	(49.1%)
T2	47	(21.4%)
NA	7	(3.1%)
Lymph node		
N0	250	(95.8%)
N1	5	(1.9%)
N2	1	(0.4%)
NA	5	(1.9%)
Stage		
Tis	42	(16.1%)
I	152	(58.2%)
IIA	54	(20.7%)
IIB	3	(1.1%)
IIIA	1	(0.4%)
NA	9	(3.4%)
Pathology		
IDC+DCIS	194	(74.3%)
ILC+LCIS	5	(1.9%)
DCIS	42	(16.1%)
Mucinous carcinoma	6	(2.3%)
Papillary carcinoma	3	(1.1%)
NA	11	(4.2%)
ER		
Positive	219	(83.9%)
Negative	34	(13.0%)
NA	8	(3.1%)
PR		
Positive	195	(74.7%)
Negative	58	(22.2%)
NA	8	(3.1%)
HER-2		
Positive	34	(13.0%)
Negative	204	(78.2%)
NA	23	(8.8%)
Ki-67		
≤14%	106	(40.6%)
>14%	90	(34.5%)
NA	65	(24.9%)
Margin		
Positive	6	(2.3%)
Negative	255	(97.7%)
Lymph node surgery		
SLNB	254	(97.3%)
SLNB+ALND	2	(0.8%)
NA	5	(1.9%)
Mean follow-up (months)	15.6±6.5 (6.9–40.4)

IDC: invasive ductal carcinoma, ILC: invasive lobular carcinoma, DCIS: ductal carcinoma in situ, ER: estrogen receptor, PR: progesteron Receptor, HER-2: human Epidermal Growth Factor Receptor 2, SLNB: sentinel lymph node biopsy, ALND: axillary lymph node dissection, NA: not available.

**Fig 2:**
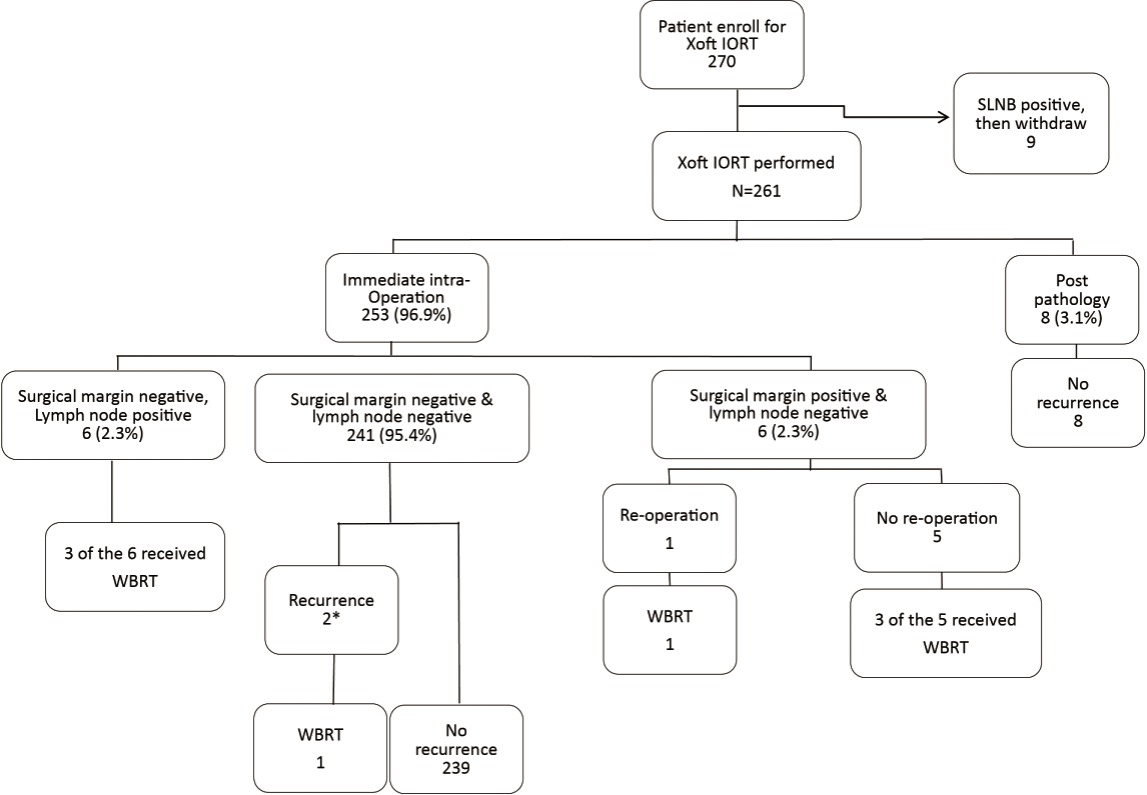
Flow chart of patients received intraoperative radiotherapy with the Xoft Axxent® eBx® delivery system. *case summary for recurrence after IORT. Case 1: 51 y/o female with right breast cancer, which was located at upper outer quadrant of breast. She received breast conserving surgery, sentinel lymph node biopsy (SLNB), and IORT. SLNB: negative for lymph node metastasis (0/2). Pathology showed DCIS, tumor size: 3 cm, ER(+, 90%), PR(+, 40%), and HER-2(+). She received adjuvant endocrine therapy with tamoxifen. Local recurrence was found at the same quadrant (right upper outer) of operated breast (tumor size: 0.6 cm, CNB: infiltrating ductal carcinoma) 1 year post surgery. Salvage simple mastectomy and SLNB were performed. Adjuvant endocrine therapy was shifted to letrozole due to hormone positive breast cancer. Case 2: 65 y/o female diagnosed with right breast cancer (CNB: DCIS (tumor size: 2.3 cm), high grade, ER(-), PR(-), HER-2(+) over upper outer quadrant. She received BCS + SLNB + IORT. Pathology showed: DCIS with microinvasion (0.1 cm), lymp node negative. She received adjuvant therapy with letrozole. Locoregional recurrence was found over right axilla (lymph node size 1 cm, CNB: IDC) 1 year post surgery. Axillary lymph node dissection was performed, and she received adjuvant chemotherapy with 4 cycles of 5-FU, lipodoxorubicin, and cyclophosphamide. Then another 4 cycles of docetaxel were given. She also received whole-breast external beam radiotherapy (WBRT) and letrozole treatment.

**Fig 3:**
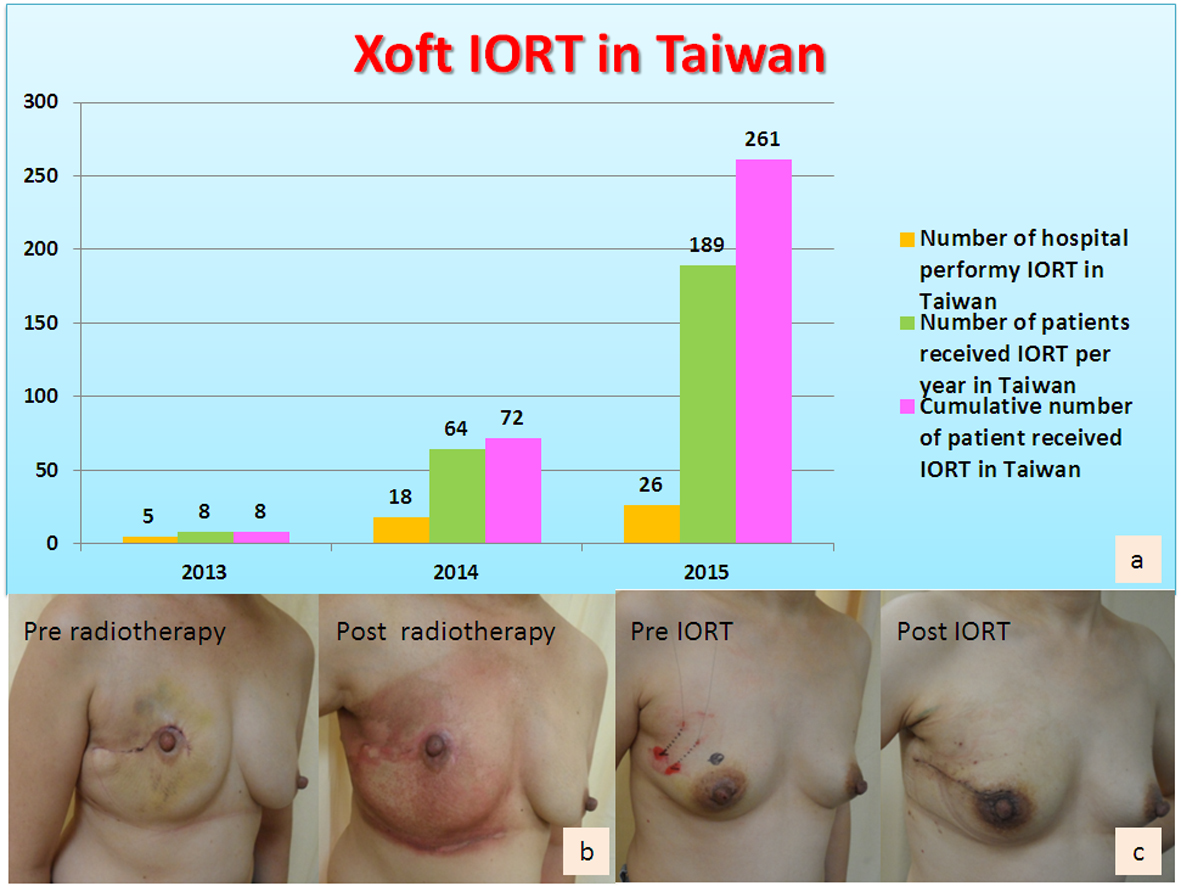
The development and application of Xoft IORT system in Taiwan. (a) The development and application of Xoft IORT system in Taiwan from 2013–2015. The T-IORTSCG comprises members from major IORT centers in Taiwan, and included 5 centers in 2013, 18 in 2014, and 26 in 2015. The number of IORT performed per year and the cumulative number of IORT performed in the past 3 years were provided. (b) Illustration of pre- and post-operative breast appearance of patients received conventional radiotherapy. (c) Illustration of pre- and post-operative breast appearance of patients received intra-operative radiotherapy.

Of those 261 patients who received IORT, 8 (3.1%) patients received WBRT (Fig 2). During a median follow up of 15.6±6.5 months, locoregional recurrence was observed in 2 (0.8%) patients (Table 2). The development and application of Xoft IORT system in Taiwan was as shown in Fig 3.

**Table 2:** Timing and types of intra-operative radiotherapy (IORT) performed.

	N = 261			
IORT dose	20 Gy irradiation			
Duration of procedure	28 ± 10 minutes (19–53)			
Duration of radiotherapy	11 minutes, (8–15)			
Xoft balloon	N = 261			
	3–4 cm spherical	30cc	178	(68.2%)
	balloon applicator	35cc	18	(6.9%)
	30–45 cc	40cc	26	(10.0%)
	n = 227 (87.0%)	45cc	4	(1.5%)
		50cc	1	(0.4%)
	4–5 cm spherical	45cc	5	(1.9%)
	balloon applicator	50cc	15	(5.7%)
	45–75 cc	55cc	2	(0.8%)
	n = 29 (11.1%)	60cc	3	(1.1%)
		70cc	3	(1.1%)
		75cc	1	(0.4%)
	NA = 5 (1.9%)		5	(1.9%)
IORT			
Timing	Immediate intra-	253	(97.0%)	
	operation		
	Post pathology	8	(3.0%)	
Indication	IORT only	253	(97.0%)	
	IORT follow by	8	(3.0%)	
	WBRT			
Lymph node	Negative	250	(95.8%)	
	Positive	6	(2.3%)	
	NA	5	(1.9%)	
Margin	Negative	255	(97.7%)	
	Positive	6	(2.3%)	
			Re-operation	5
			No re-operation	1
Locoregional recurrence	No	259	(99.2%)	
	Yes	2	(0.8%)	
Mortality	No	261	(100%)	
	Yes	0	(0%)	

IORT: intra-operative radiotherapy, WBRT: whole-breast external beam radiotherapy, NA: not available.

The criteria used by the participating hospitals to select patients for IORT were compared with those used in the ELIOT [7] and TARGIT-A [8] studies, and summarized in Table 3.

**Table 3:** Comparison of patients selection criteria of Xoft IORT in Taiwan with ELIOT and TARGIT-A trials.

Indication for IORT	ELIOT trial^7^			TARGIT-A trial^8^			T-IORTSCG			P value
Age (years)	48–49	44	(7%)	<45	17/1113	(2%)	Mean 52.9 ± 9.8			<0.01
	50–59	286	(44%)	45–54	212/1113	(19%)	<45	43	(16.5%)	
	60–69	259	(40%)	55–64	443/1113	(40%)	45–60	147	(56.3%)	
	≥70	62	(10%)	65–74	355/1113	(32%)	>60	56	(21.5%)	
				>74	86/1113	(8%)	NA	15	(5.7%)	
Tumor size	≤1 cm	199	(31%)	<1 cm	381/1056	(36%)	Tumor Size (in situ, cm)			<0.01
	1–1.5cm	243	(38%)	1-2cm	531/1056	(50%)	1.2 ± 0.75 (0.15–3.0)			
	1.5-2cm	120	(19%)	>2 cm	144/1056	(14%)	Tumor Size (invasive, cm)			
	>2 cm	83	(13%)	Unknow	57/1113	(5%)	1.49 ± 0.77 (0.1–4.2)			
							T1a	18	(8.2%)	
							T1b	40	(18.2%)	
							T1c	108	(49.1%)	
	***Poor prognosis if tumor >2cm***						T2	47	(21.4%)	
							NA	7	(3.1%)	
Lymph node	None	478	(74%)	0	866/1059	(82%)	N0	242	(92.7%)	<0.01
status	1–3	138	(21%)	1–3	155/1059	(15%)	N1	12	(4.6%)	
	≥ 4	31	(5%)	>3	38/1059	(4%)	N2	1	(0.4%)	
	***Poor prognosis if > 4 nodes metastasis***			Unknow	54/1113	(5%)	NA	6	(2.3%)	
Histology	Ductal	524	(81%)	Invasive ductal carcinoma			IDC+DCIS	194	(74.3%)	<0.01
	Lobular	53	(8%)		1012/1070	(95%)	ILC+LCIS	5	(1.9%)	
	Ductal and lobular			Invasive lobular carcinoma			DCIS	42	(16.1%)	
		17	(3%)		47/1070	(4%)	Mucinous cancer 6		(2.3%)	
	Other	53	(8%)	Mixed	32/1070	(3%)	Papillary cancer 3		(1.1%)	
				Unknow	43/1113	(4%)	NA	11	(4.2%)	
Grade	G1	196	(31%)	1	341/1040	(33%)				0.02
	G2	305	(48%)	2	540/1040	(52%)				
	G3	129	(20%)	3	159/1040	(15%)				
				Unknow	73/1113	(7%)				
	***Poor prognosis if >G3***									
ER	Negarive 63		(10%)	Oestrogen-receptor positive			ER			0.16
	Positive 583		(90%)		962/1063	(90%)	Positive	219	(83.9%)	
				Oestrogen-receptor negative			Negative	34	(13.0%)	
					101/1063	(10%)	NA	8	(3.1%)	
				Oestrogen-receptor status						
				unknow	50/1113	(4%)				
PR	Negative 158		(24%)				PR			0.62
	Positive 487		(76%)				Positive	195	(74.7%)	
							Negative	58	(22.2%)	
							NA	8	(3.1%)	
HER-2				HER-2 (ERBBB2) recepter status			HER-2			0.70
				Positive	132/991	(13%)	Positive	34	(13.0%)	
				Negative	859/991	(87%)	Negative	204	(78.2%)	
				Not done	31/1113	(3%)	NA	23	(8.8%)	
				Unknow	91/1113	(8%)				
Ki-67	<14%	263	(41%)				≤14%	106	(40.6%)	<0.01
	14–20%	138	(21%)				>14%	90	(34.5%)	
	>20%	244	(38%)				NA	65	(24.9%)	

T-IORTSCG: Taiwan IORT study cooperative group, IDC: invasive ductal carcinoma, ILC: invasive lobular carcinoma, DCIS: ductal carcinoma in situ, ER: estrogen receptor, PR: progesteron Receptor, HER-2:human Epidermal Growth Factor Receptor 2, NA: not available.

The clinical and pathologic manifestations of patients received IORT in current study differed markedly from those used in the ELIOT and TARGIT-A studies. Specifically, patients selected for IORT in Taiwan tended to be younger (16.5% <45 y/o in T-IORTSCG, 7% 48–49 y/o in ELIOT, and 2% < 45 y/o in TARGIT-A, P<0.01), their tumors tended to be larger (T2 tumor 21.4% in T-IORTSCG compared to 13% in ELIOT, and 14% in TARGIT-A, P<0.01), the prevalence of lymph node metastasis tended to be lower (92.7% node negative in T-IORTSCG compared to 74% in ELIOT, and 82% in TARGIT-A, P<0.01).

## Discussion

Targeted radiation can be delivered to the tumor bed intraoperatively by a number of energy sources. ELIOT involves administering electrons in one session during surgery with a total dose of 21 Gy [7, 12]. The Intrabeam device, which was used in the TARGIT-A trial [8, 9], is a miniature electron beam-driven X-ray source that provides a point source of low-energy X-rays (50 kV maximum) at the tip of a 3.2-mm diameter tube. The Axxent eBX system, which was used in the current study, is an alternative to radioactive-isotope based therapy [11]. eBX utilizes a miniature X-ray source to deliver high-dose radiation to the target area at low energy, thus obviating the need for a highly shielded environment [11]. The system, which received Food and Drug Administration (FDA) approval for the treatment of breast cancer in January 2006 [11], is a relatively new method of delivering APBI and aims to replace WBRT in women suitable for BCS. As shown in Fig 3, the rapid increase in the number of hospitals in Taiwan that have adopted the Xoft Axxent® eBx® system for IORT is an evidence for its wide acceptance among surgeons and radiation oncologists as a treatment modality for women who are eligible for BCS.

A number of clinical trials have provided evidence that IORT is an efficacious treatment modality [7–9, 11, 12]. However, the indications for IORT are not well defined and varied among trials. As young age is viewed as a poor prognostic factor for disease recurrence [12], the criteria for suggestion of age for patients to receive IORT was not clear defined. According to the recent ASTRO guidelines [13] and the GEC-ESTRO working group recommends [14], partial-breast irradiation should be attempted in women greater than 50 years. The age criteria was a minimum of 45 year-old or older in TARGIT-A and other study [9, 11], or aged 48–75 years in ELIOT [7]. Although the mean age in our study was 52.9 ± 9.8, 16.5% of the patients were younger than 45 years. This may be reflected that young female has higher motivation to decrease the frequency of visit to hospital, and economy more independent to afford the fee of IORT (cost about $8000 US dollars in Taiwan), which was not reimbursed by our national insurance. In ELIOT [7] and GEC-ESTRO [20] trials, age was not a poor prognostic factor for disease local recurrence. However, the safety of younger (age less than 45) patients to receive IORT should be caution.

Most trials agreed that IORT is most appropriate for women with unifocal disease detected on conventional breast images [9, 11], and MRI was not mandatory. However, the upper limitation of tumor size has not been determined. In the ELIOT trial it was found that IORT was effective for small tumors with a maximum tumor diameter of 2.5 cm suitable for BCS [7]. In the study by Ivanov et al, IORT was determined to be appropriate for tumors measuring less than 3 cm [11], and in the TARGIT-A trial, the therapy was shown to be effective for any tumor suitable for wide local excision [9]. Currently, patients selected to receive IORT were suggestive to have smaller tumor (≤2 cm) to prevent local recurrence [7].

Lymph node status was not strictly regulated in either the ELIOT or TARGIT-A trial, but node negative status was a criterion in some study [11]. Lymph node metastasis is regarded as the most important poor prognostic factor [21]. It remains unclear, however, whether lymph node metastasis is a contraindication for IORT. In the ELIOT trial, four or more positive nodes were associated with poorer prognosis [7]. In that trial, 21% of patients who received IORT had 1–3 positive lymph nodes and in the TARGIT-A trial [8] 15% of patients had 1–3 positive lymph nodes (Table 3). Based on those findings, low burden axillary disease (<3 positive nodes) is not a contraindication for IORT [7, 12]. In meta-analysis [22], adjuvant radiation of regional nodes for node positive breast cancer have shown an improvement in overall survival. Patients who received IORT with positive lymph nodes, either diagnosed before IORT or found after final pathologic check-up, should be discussed whether further radiotherapy would be needed.

In the TARGIT-A trial [8, 9], only patients with histological diagnosis of IDC were selected to receive IORT whereas in other study [11], patients with either IDC or DCIS were recruited. Preoperative histologic diagnosis of lobular carcinoma was a criterion for exclusion in the TARGIT-A trial [9] and other study [11] as lobular tumors are associated with a higher propensity for being multifocal [23, 24]. However, in the ELIOT trial, lobular histology was neither a poor prognostic factor nor a contraindication for IORT [7]. Most trials [7, 8, 11] and guidelines [13, 14] do not include neoadjuvant chemotherapy as an indication for IORT or partial breast irradiation. Whether IORT is appropriate for patients with DCIS is unclear? In ASTRO and GEC-ESTRO guideline, APBI is not recommended as treatment for pure DCIS [13, 14]. However, in recent published GEC-ESTRO trial, 6% of APBI patients were pure DCIS [20]. In our study, 16.8% of patients who received IORT had pure DCIS lesions. Whether patients with pure DCIS lesions should receive IORT as adjuvant radiotherapy to prevent local recurrence remains unclear and requires further study.

In our current study, 2 (0.8%) patients were found to have locoregional recurrences (one patient found to have local recurrence in the same quadrant of operated breast, and the other with regional recurrence at the axilla) in the mean 15.6 months follow-up period (Fig 2). The 5-year local recurrence rate was 4.4% in ELIOT, and 3.3% in TARGIT-A trials. According to the results of the ELIOT trial, patients with disease characteristics associated with local recurrence such as tumor size greater than 2 cm, tumor of grade 3, four of more positive nodes, and triple-negative tumors should not be treated with IORT alone [7]. The 2 patients, who diagnosed to have locoregional recurrence in the conserved breast (or axilla) in our study, were found within 1.5 year post operation. The new diagnosed breast cancer lesion could not to be sure to be a “recurrence” after BCS followed by Xoft IORT or a “pre-existing multifocal or multicentric breast cancer lesion” found shortly after treatment. It might be reasonable that incorporating MRI into pre-operative screening could reduce the possibility of enroll “occult multi-focal or multicentric breast cancer patients” [25], and therefore decrease the early “recurrence”.

In our study, 8(3.1%) patients who received IORT via the Xoft Axxent® eBx® system required WBRT. Three of them due to positive lymph node metastasis, one due to local recurrence, and the other four due to positive surgical margin (Fig 2). In the TARGIT-A trial, 15.2% of patients required supplemental WBRT after TARGIT [8]. As the concept of risk adapted IORT, it is recommended that supplemental WBRT be administered to patients who present with tumor-free margins smaller than 1 mm, extensive in-situ components, or unexpected invasive lobular carcinoma [8].

In the current study we investigated the indications for and clinical outcomes associated with the delivery of IORT via the Xoft Axxent® eBx® IORT system in patients with primary operable breast cancer at medical centers in Taiwan during the period of 2013–2015. Our analysis revealed that the selection criteria used by the participating hospitals in this study differed markedly from those used in the ELIOT and TARGIT-A studies. Specifically, patients selected for IORT in Taiwan tended to be younger, their tumors tended to be larger and the prevalence of lymph node metastasis tended to be lower. Limitations in this study include its retrospective nature and possible selection bias. The lack of long-term follow-up results in the current study precluded us from determining whether the Xoft Axxent® eBx® system results in adequate local disease control. However, current study did provide important information for patients receiving IORT with Xoft Axxent® eBx® system in a real world experience, which was derived from a national population based database.

In conclusion, the Xoft Axxent® eBx® system is well-accepted by physicians and patients in Taiwan. The characteristics of patients selection in our study might reflected the need of patients desired for IORT. Our findings together with those from previous studies should help to delineate the role and value of this new adjuvant radiotherapy technique in the field of breast cancer.

## Supporting information

S1 Table Detailed patient information of our study. (XLS)

## References

[1] Veronesi U, Cascinelli N, Mariani L, Greco M, Saccozzi R, Luini A, et al Twenty-year follow-up of a randomized study comparing breast-conserving surgery with radical mastectomy for early breast cancer. N Engl J Med. 2002;347(16):1227–32. doi: 10.1056/NEJMoa020989 12393819 10.1056/NEJMoa020989

[2] Fisher B, Anderson S, Bryant J, Margolese RG, Deutsch M, Fisher ER, et al Twenty-year follow-up of a randomized trial comparing total mastectomy, lumpectomy, and lumpectomy plus irradiation for the treatment of invasive breast cancer. N Engl J Med. 2002;347(16):1233–41. doi: 10.1056/NEJMoa022152 12393820 10.1056/NEJMoa022152

[3] Clarke M, Collins R, Darby S, Davies C, Elphinstone P, Evans V, et al Effects of radiotherapy and of differences in the extent of surgery for early breast cancer on local recurrence and 15-year survival: an overview of the randomised trials. Lancet. 2005;366(9503):2087–106. doi: 10.1016/S0140-6736(05)67887-7 16360786 10.1016/S0140-6736(05)67887-7

[4] Farrow DC, Hunt WC, Samet JM. Geographic variation in the treatment of localized breast cancer. N Engl J Med. 1992;326(17):1097–101. doi: 10.1056/NEJM199204233261701 1552910 10.1056/NEJM199204233261701

[5] Dolan JT, Granchi TS, Miller CC 3rd, Brunicardi FC. Low use of breast conservation surgery in medically indigent populations. Am J Surg. 1999;178(6):470–4. 10670855 10.1016/s0002-9610(99)00226-3

[6] Hahn CA, Marks LB, Chen DY, Lind PA, Lind HM, Prosnitz LR. Breast conservation rates-barriers between tertiary care and community practice. Int J Radiat Oncol Biol Phys. 2003;55(5):1196–9. 12654427 10.1016/s0360-3016(02)04475-9

[7] Veronesi U, Orecchia R, Maisonneuve P, Viale G, Rotmensz N, Sangalli C, et al Intraoperative radiotherapy versus external radiotherapy for early breast cancer (ELIOT): a randomised controlled equivalence trial. Lancet Oncol. 2013;14(13):1269–77. doi: 10.1016/S1470-2045(13)70497-2 24225155 10.1016/S1470-2045(13)70497-2

[8] Vaidya JS, Wenz F, Bulsara M, Tobias JS, Joseph DJ, Keshtgar M, et al Risk-adapted targeted intraoperative radiotherapy versus whole-breast radiotherapy for breast cancer: 5-year results for local control and overall survival from the TARGIT-A randomised trial. Lancet. 2014;383(9917):603–13. doi: 10.1016/S0140-6736(13)61950-9 24224997 10.1016/S0140-6736(13)61950-9

[9] Vaidya JS, Joseph DJ, Tobias JS, Bulsara M, Wenz F, Saunders C, et al Targeted intraoperative radiotherapy versus whole breast radiotherapy for breast cancer (TARGIT-A trial): an international, prospective, randomised, non-inferiority phase 3 trial. Lancet. 2010;376(9735):91–102. doi: 10.1016/S0140-6736(10)60837-9 20570343 10.1016/S0140-6736(10)60837-9

[10] Mehta VK, Algan O, Griem KL, Dickler A, Haile K, Wazer DE, et al Experience with an electronic brachytherapy technique for intracavitary accelerated partial breast irradiation. Am J Clin Oncol. 2010;33(4):327–35. doi: 10.1097/COC.0b013e3181d79d9e 20375833 10.1097/COC.0b013e3181d79d9e

[11] Ivanov O, Dickler A, Lum BY, Pellicane JV, Francescatti DS. Twelve-month follow-up results of a trial utilizing Axxent electronic brachytherapy to deliver intraoperative radiation therapy for early-stage breast cancer. Ann Surg Oncol. 2011;18(2):453–8. doi: 10.1245/s10434-010-1283-x 20737219 10.1245/s10434-010-1283-x

[12] Veronesi U, Orecchia R, Luini A, Galimberti V, Zurrida S, Intra M, et al Intraoperative radiotherapy during breast conserving surgery: a study on 1,822 cases treated with electrons. Breast Cancer Res Treat. 2010;124(1):141–51. doi: 10.1007/s10549-010-1115-5 20711810 10.1007/s10549-010-1115-5

[13] Smith BD, Arthur DW, Buchholz TA, Haffty BG, Hahn CA, Hardenbergh PH, et al Accelerated partial breast irradiation consensus statement from the American Society for Radiation Oncology (ASTRO). Int J Radiat Oncol Biol Phys. 2009;74(4):987–1001. doi: 10.1016/j.ijrobp.2009.02.031 19545784 10.1016/j.ijrobp.2009.02.031

[14] Guedea F, Venselaar J, Hoskin P, Hellebust TP, Peiffert D, Londres B, et al Patterns of care for brachytherapy in Europe: updated results. Radiother Oncol. 2010;97(3):514–20. doi: 10.1016/j.radonc.2010.09.009 20950878 10.1016/j.radonc.2010.09.009

[15] Giuliano AE, McCall L, Beitsch P, Whitworth PW, Blumencranz P, Leitch AM, et al Locoregional recurrence after sentinel lymph node dissection with or without axillary dissection in patients with sentinel lymph node metastases: the American College of Surgeons Oncology Group Z0011 randomized trial. Ann Surg. 2010;252(3):426–32; discussion 32–3. doi: 10.1097/SLA.0b013e3181f08f32 20739842 10.1097/SLA.0b013e3181f08f32 PMC5593421

[16] Anderson BO, Masetti R, Silverstein MJ. Oncoplastic approaches to partial mastectomy: an overview of volume-displacement techniques. Lancet Oncol. 2005;6(3):145–57. doi: 10.1016/S1470-2045(05)01765-1 15737831 10.1016/S1470-2045(05)01765-1

[17] Holmes DR, Schooler W, Smith R. Oncoplastic approaches to breast conservation. Int J Breast Cancer. 2011;2011:303879 doi: 10.4061/2011/303879 22295216 10.4061/2011/303879 PMC3262568

[18] Goldhirsch A, Winer EP, Coates AS, Gelber RD, Piccart-Gebhart M, Thurlimann B, et al Personalizing the treatment of women with early breast cancer: highlights of the St Gallen International Expert Consensus on the Primary Therapy of Early Breast Cancer 2013. Ann Oncol. 2013;24(9):2206–23. doi: 10.1093/annonc/mdt303 23917950 10.1093/annonc/mdt303 PMC3755334

[19] Network NCC. NCCN Clinical Practice Guidelines in Oncology-Breast Cancer Fort Washington, PA: National Comprehensive Cancer Network; 2014 Available from: http://www.nccn.org/professionals/physician_gls/PDF/breast.pdf.

[20] Strnad V, Ott OJ, Hildebrandt G, Kauer-Dorner D, Knauerhase H, Major T, et al 5-year results of accelerated partial breast irradiation using sole interstitial multicatheter brachytherapy versus whole-breast irradiation with boost after breast-conserving surgery for low-risk invasive and in-situ carcinoma of the female breast: a randomised, phase 3, non-inferiority trial. Lancet. 2016;387(10015):229–38. doi: 10.1016/S0140-6736(15)00471-7 26494415 10.1016/S0140-6736(15)00471-7

[21] Cianfrocca M, Goldstein LJ. Prognostic and predictive factors in early-stage breast cancer. Oncologist. 2004;9(6):606–16. doi: 10.1634/theoncologist.9-6-606 15561805 10.1634/theoncologist.9-6-606

[22] Budach W, Bolke E, Kammers K, Gerber PA, Nestle-Kramling C, Matuschek C. Adjuvant radiation therapy of regional lymph nodes in breast cancer—a meta-analysis of randomized trials- an update. Radiat Oncol. 2015;10:258 doi: 10.1186/s13014-015-0568-4 26691175 10.1186/s13014-015-0568-4 PMC4687086

[23] Derias M, Subramanian A, Allan S, Shah E, Teraifi HE, Howlett D. The Role of Magnetic Resonance Imaging in the Investigation and Management of Invasive Lobular Carcinoma-A 3-Year Retrospective Study in Two District General Hospitals. Breast J. 2016;22(4):384–9. doi: 10.1111/tbj.12594 27265271 10.1111/tbj.12594

[24] Christgen M, Steinemann D, Kuhnle E, Langer F, Gluz O, Harbeck N, et al Lobular breast cancer: Clinical, molecular and morphological characteristics. Pathol Res Pract. 2016;212(7):583–97. doi: 10.1016/j.prp.2016.05.002 27233940 10.1016/j.prp.2016.05.002

[25] Lai HW, Chen CJ, Lin YJ, Chen SL, Wu HK, Wu YT, et al Does Breast Magnetic Resonance Imaging Combined With Conventional Imaging Modalities Decrease the Rates of Surgical Margin Involvement and Reoperation?: A Case-Control Comparative Analysis. Medicine (Baltimore). 2016;95(22):e3810 doi: 10.1097/MD.0000000000003810 27258520 10.1097/MD.0000000000003810 PMC4900728

